# Associations of polygenic risk scores with posttraumatic stress symptom trajectories following combat deployment

**DOI:** 10.1017/S0033291723000211

**Published:** 2023-10

**Authors:** Laura Campbell-Sills, Santiago Papini, Sonya B. Norman, Karmel W. Choi, Feng He, Xiaoying Sun, Ronald C. Kessler, Robert J. Ursano, Sonia Jain, Murray B. Stein

**Affiliations:** 1 Department of Psychiatry, University of California San Diego, La Jolla, CA, USA; 2 Division of Research, Kaiser Permanente Northern California, Oakland, CA, USA; 3 Executive Division, National Center for PTSD, White River Junction, VT, USA; 4 VA Center of Excellence for Stress and Mental Health, San Diego, CA, USA; 5 Department of Psychiatry, Center for Precision Psychiatry, Massachusetts General Hospital, Boston, MA, USA; 6 Psychiatric and Neurodevelopmental Genetics Unit, Center for Genomic Medicine, Massachusetts General Hospital, Boston, MA, USA; 7 Stanley Center for Psychiatric Research, Broad Institute, Boston, MA, USA; 8 Herbert Wertheim School of Public Health and Human Longevity Science, University of California San Diego, La Jolla, CA, USA; 9 Department of Health Care Policy, Harvard Medical School, Boston, MA, USA; 10 Center for the Study of Traumatic Stress, Department of Psychiatry, Uniformed Services University of the Health Sciences, Bethesda, MD, USA; 11 Psychiatry Service, VA San Diego Healthcare System, San Diego, CA, USA

**Keywords:** Latent growth mixture modeling, military personnel, polygenic risk scores, posttraumatic stress, trauma

## Abstract

**Background:**

Identification of genetic risk factors may inform the prevention and treatment of posttraumatic stress disorder (PTSD). This study evaluates the associations of polygenic risk scores (PRS) with patterns of posttraumatic stress symptoms following combat deployment.

**Method:**

US Army soldiers of European ancestry (*n* = 4900) provided genomic data and ratings of posttraumatic stress symptoms before and after deployment to Afghanistan in 2012. Latent growth mixture modeling was used to model posttraumatic stress symptom trajectories among participants who provided post-deployment data (*n* = 4353). Multinomial logistic regression models tested independent associations between trajectory membership and PRS for PTSD, major depressive disorder (MDD), schizophrenia, neuroticism, alcohol use disorder, and suicide attempt, controlling for age, sex, ancestry, and exposure to potentially traumatic events, and weighted to account for uncertainty in trajectory classification and missing data.

**Results:**

Participants were classified into low-severity (77.2%), increasing-severity (10.5%), decreasing-severity (8.0%), and high-severity (4.3%) posttraumatic stress symptom trajectories. Standardized PTSD-PRS and MDD-PRS were associated with greater odds of membership in the high-severity *v.* low-severity trajectory [adjusted odds ratios and 95% confidence intervals, 1.23 (1.06–1.43) and 1.18 (1.02–1.37), respectively] and the increasing-severity *v.* low-severity trajectory [1.12 (1.01–1.25) and 1.16 (1.04–1.28), respectively]. Additionally, MDD-PRS was associated with greater odds of membership in the decreasing-severity *v.* low-severity trajectory [1.16 (1.03–1.31)]. No other associations were statistically significant.

**Conclusions:**

Higher polygenic risk for PTSD or MDD is associated with more severe posttraumatic stress symptom trajectories following combat deployment. PRS may help stratify at-risk individuals, enabling more precise targeting of treatment and prevention programs.

## Introduction

Traumatic events affect most adults during their lifetimes (Benjet et al., 2016), yet there is marked heterogeneity in the type, severity, and course of psychological symptoms that result. The modal response to trauma is resilience, wherein only minimal or transient symptoms emerge following the stressful event (Bonanno, 2004; Galatzer-Levy, Huang, & Bonanno, 2018). On the other end of the severity spectrum is posttraumatic stress disorder (PTSD), a condition characterized by re-experiencing, avoidance, negative alterations in cognition or mood, and hypervigilance that persists for 3 months or more (American Psychiatric Association, 2013). Approximately 5–10% of trauma-exposed individuals develop PTSD, with many others suffering from acute stress reactions lasting less than 3 months, or from clinically significant symptoms that fail to meet full PTSD diagnostic criteria (Bryant, 2019; Yehuda et al., 2015).

Gaining insight into the determinants of posttraumatic outcomes requires investigation of a wide range of individual differences, trauma characteristics, and social factors (Brewin, Andrews, & Valentine, 2000; Daskalakis, Rijal, King, Huckins, & Ressler, 2018; Nemeroff et al., 2006; Tolin & Foa, 2006); and understanding the role genetic factors play is part of this effort. Evidence from genome-wide association studies (GWAS) implicates multiple risk loci in the etiology of PTSD (Duncan et al., 2018; Nievergelt et al., 2019; Stein et al., (2016, 2021b; Xie et al., 2013), the effects of which can be pooled and expressed as polygenic risk scores (PRS). Studies have begun to examine if PRS are associated with PTSD diagnosis and symptom severity among individuals with potential trauma exposure (Misganaw et al., 2019; Tamman et al., 2022; Waszczuk et al., 2020). Consistent with other evidence of shared genetic underpinnings of mental disorders (Bulik-Sullivan et al., 2015; Gandal et al., 2018), PRS for PTSD diagnosis/symptom severity, as well as PRS for related disorders and traits (e.g. depression, neuroticism), have demonstrated associations with PTSD phenotypes (Misganaw et al., 2019; Tamman et al., 2022; Waszczuk et al., 2020).

Early suggestions that PRS hold promise as a risk stratification tool are encouraging but require replication and extension to other populations and settings. Military personnel are disproportionally impacted by PTSD (Fulton et al., 2015), due in part to the nature and frequency of trauma exposure within this population. The Pre/Post Deployment Study (PPDS) of the Army Study to Assess Risk and Resilience in Servicemembers (Army STARRS; Kessler et al., 2013; Ursano et al., 2014) provides an opportunity to investigate whether PRS are associated with posttraumatic stress symptoms in combat-deployed military personnel. Importantly, the PPDS included multiple outcome assessments during the period following potential trauma exposure (approximately 1, 3, and 9 months after return from deployment), permitting investigation of posttraumatic stress symptoms using a dynamic perspective that considers both severity and time course of symptoms.

Several patterns of posttraumatic stress symptoms have been observed in longitudinal studies of trauma-exposed samples, which are distinguished based on a combination of severity and time course. Patterns reported consistently across samples include trajectories characterized by low stable symptoms (typically labeled ‘resilient’), high symptoms after exposure that remit quickly (‘recovery’), low symptoms after exposure that later worsen to clinically significant levels (‘delayed onset’), and chronically elevated symptoms (‘chronic’; Bonanno et al., 2008; Fan, Long, Zhou, Zheng, & Liu, 2015; Galatzer-Levy et al., 2018; Lowe et al., 2021; Pietrzak et al., 2014; Wang et al., 2018). These findings raise important questions beyond the basic issue of whether PRS are associated with more severe posttraumatic stress symptoms or PTSD diagnosis. These questions include whether PRS may help identify at-risk individuals who would be difficult to detect using other clinical tools (e.g. those with a delayed onset of symptoms) and whether PRS may help differentiate individuals vulnerable to short-term posttraumatic stress symptoms from those at risk of chronic symptoms. Evidence of either of these capabilities would increase the potential value of PRS for targeted prevention efforts.

Using genetic data from PPDS participants, we evaluated whether a PRS for PTSD predicted posttraumatic stress symptom trajectories after combat deployment. We hypothesized that higher PTSD-PRS would be associated with more severe trajectories. Given prior evidence of the cross-disorder predictive capabilities of PRS, we also expected that other psychiatric PRS would be associated with patterns of posttraumatic stress symptoms. Genetic vulnerability to PTSD has been found to covary with genetic risk for depression, schizophrenia, and neuroticism (Nievergelt et al., 2019). Additionally, alcohol misuse and PTSD are highly comorbid (Thomas et al., 2010), and may have partly overlapping neurobiological etiology (Suh & Ressler, 2018). Findings suggestive of genetic links between PTSD and suicidality have also been reported (Daskalakis et al., 2021). Based on these findings, we also investigated whether PRS for major depressive disorder (MDD), schizophrenia, neuroticism, alcohol use disorder (AUD), and suicide attempt were associated with post-deployment trajectories of posttraumatic stress symptoms.

## Method

### Participants

Participants were from the PPDS component of Army STARRS (Kessler et al., 2013; Ursano et al., 2014), which recruited 9488 soldiers from three Brigade Combat Teams (BCTs) during 2012, 1–2 months before the teams deployed to Afghanistan. Due to the limited availability of reference GWAS data in other populations, we limited our analyses to the subsample of 4900 soldiers of genetically determined European ancestry with a mean age of 25.9 years (s.d. = 5.9), who were mostly males (94.5%). Among the participants of European ancestry, 546 (11.14%) could not be directly included in the trajectory analyses because they did not complete any of the post-deployment assessments of posttraumatic stress symptoms. However, we accounted for these participants using inverse probability of response weighting, which is a robust approach for incorporating missing data in the estimates of effects that is less biased than ‘completers-only’ analyses (described in ‘Data analysis’ section).

### Polygenic risk scores

We used published summary statistics from GWAS of PTSD (Stein et al., 2021b), MDD (Howard et al., 2019), schizophrenia (Ripke, Walters, & O'Donovan, 2020), AUD (Walters et al., 2018), and suicide attempt (Mullins et al., 2022) phenotypes and a European ancestry reference panel to estimate single-nucleotide polymorphism effect sizes with PRS-CS-auto (Ge, Chen, Ni, Feng, & Smoller, 2019) and summed with PLINK 2.0 (Chang et al., 2015) to create PRS, which were standardized within the sample for subsequent analyses. More details about the data sources for the summary statistics used to calculate PRS for each phenotype are provided in online Supplementary Table S1. The methods for DNA collection, genotyping, quality control, and ancestry assignment are reported in a previous publication (Stein et al., (2016).

### Measures

All PPDS surveys are available online (https://starrs-ls.org/#/page/instruments). Exposure to potentially traumatic events (PTEs) was assessed across all four waves: pre-deployment (1–2 months before deployment), post-deployment (within 2–3 weeks of returning from deployment), and two follow-ups (at 2–3 months and 8–9 months after return from deployment). At pre-deployment, participants who had previous deployments (54.2%) completed a checklist of 15 PTEs that may have occurred during deployment (e.g. combat, unit casualties, witnessing death and destruction). Additionally, all participants completed a checklist of 15 lifetime PTEs (e.g. physical assault, sexual assault, life-threatening illness or injury), excluding any experiences that had already been reported as deployment PTEs. Two binary variables (yes/no) were created from these checklists that denoted PTE exposure (deployment/lifetime). In the full sample, 47.3% reported at least one PTE from previous deployments, and 80.6% reported at least one (non-deployment) lifetime PTE. Participants who completed at least one survey after returning from the index deployment (*n* = 4354) also completed a checklist of exposure to PTEs from that recent deployment. Soldiers who participated in the post-deployment survey completed a 16-item PTE checklist; an abridged 6-item version of this checklist was administered in the follow-up surveys for any participant who had not provided this information in an earlier survey. Using all available data from the post-deployment and follow-up surveys, we created binary (yes/no) exposure variables for six distinct PTEs during the index deployment. The prevalence of these PTEs were 80.5% (combat patrols), 73.0% (fire rounds at or take fire from enemy), 9.5% (wounded), 70.9% (unit member killed or wounded), 67.0% (exposure to severely wounded or dead people), and 83.7% (other highly stressful experience). Finally, in the two follow-up surveys participants completed a checklist of 12 PTEs that occurred since their return from the index deployment; 40.3% reported at least one new PTE. PTE variables based on experiences that occurred prior to the first time-point in the trajectory analyses were used as time-invariant covariates in the analysis of PRS associations with trajectories; PTE variables collected after return from deployment were used to aid in the interpretation of trajectories. We describe this further in the ‘Data analysis’ section.

Posttraumatic stress symptom severity was assessed at all waves using items from the PTSD Checklist, civilian version (PCL-C; Weathers, Litz, Herman, Huska, & Keane, 1993). Participants were asked to respond to PCL-C items in reference to *any* stressful experience. The two follow-ups assessed all DSM-IV PTSD symptoms; however, the pre-deployment survey evaluated six symptoms (intrusive recollections, physiological reactivity to reminders, avoidance of thoughts/feelings, avoidance of situations, difficulty concentrating, and exaggerated startle), and the post-deployment survey evaluated five symptoms (intrusive recollections, physiological reactivity to reminders, sense of foreshortened future, difficulty concentrating, and exaggerated startle). At the follow-ups where all PCL-C items were administered, the total score based on all items and the scores based on the reduced-item versions of the PCL-C were highly correlated (all *r*s > 0.95; online Supplementary Table S2), which suggests a high degree of concordance. To harmonize total scores on these PCL-C measures, respondents' severity ratings of each posttraumatic stress symptom (0–4; ‘not at all’ to ‘extremely’ bothered) at a given time point were summed, then the percentage of the maximum possible total score at that assessment point was calculated.

We selected the post-deployment assessment as the starting point of our trajectory analyses (described in ‘Data analysis’ section). This allowed us to model the course of posttraumatic stress symptom trajectories after exposure to PTEs during the index deployment. Additionally, length of deployment differed across the three BCTs, making the time from pre- to post-deployment assessments highly variable (BCT 1: *M* = 7.24, s.d. = 0.67; BCT 2: *M* = 10.12, s.d. = 0.84; BCT 3: *M* = 10.36, s.d. = 0.42), which would have limited interpretability of the trajectories.

### Data analysis

We adhered to the guidelines for reporting on latent trajectory studies (van de Schoot, Sijbrandij, Winter, Depaoli, & Vermunt, 2017); the completed 21-item checklist is provided in online Supplementary Table S3. Trajectory analyses were conducted in R (R Core Team, 2022) with the *lcmm* package (version 1.9.4; Proust-Lima, Philipps, & Liquet, 2017), which uses maximum-likelihood estimation to include participants with data assumed to be missing at random. We examined modeling approaches that varied in their specifications of within-class heterogeneity: latent growth mixture models (LGMM; within-trajectory variation in the intercepts and/or slopes is allowed) and latent class growth analysis (within-trajectory variance of the intercepts and slopes are fixed to zero). We compared models with one to five trajectories and selected a final model based on sample size-adjusted Bayesian information criteria (SSA-BIC; lower is better), entropy (reflects classification accuracy; values closer to 1 are better), and the Lo–Mendell–Rubin-likelihood ratio test (LMR-LRT; significant value indicates better fit). Parsimonious models were favored over models that improved fit by adding an insufficiently distinct trajectory that included a low proportion of participants. The variance–covariance matrix of the random effects was constrained to be equal across trajectories to facilitate model convergence. Random starting values for the multi-trajectory models used information from the single-trajectory model; values were generated using gridsearch with 100 repetitions and a maximum of 100 iterations in the optimization algorithm. Analysis outputs with precise information about the number of random start values and final iterations included are provided in the online Supplementary material.

Since three post-deployment assessments were collected, we were able to fit linear (but not quadratic) trajectories. Participants with at least one PCL assessment in the post-deployment and follow-up phase were used to estimate posttraumatic stress trajectories (*n* = 4354). Among these, 11.0% were missing two assessments, 29.5% were missing one assessment, and the majority (59.4%) had all three assessments. Exclusion of participants that are missing assessments (e.g. a ‘completers only’ approach) can lead to selection bias and inaccurate characterization of trajectories; therefore, these analyses estimate a probability of trajectory assignment for each participant that had data for at least one assessment. In other words, rather than exclude a participant whose trajectory is unclear because of missing data, the probability of that participant's predicted trajectory is adjusted to account for this uncertainty.

A non-monotonic missing data pattern was observed such that only a minority of participants (3.5%) completed the post-deployment assessment but did not return for any follow-ups. Pre-deployment posttraumatic stress symptom severity was significantly higher among participants with missing data at post-deployment (*M* difference = 3.50, *p* < 0.001) and at the first follow-up (*M* difference = 3.03, *p* < 0.001), but not the last follow-up (*M* difference = −0.48, *p* = 0.37). Given these associations, pre-deployment posttraumatic stress symptom severity was included as a covariate in the trajectory analyses to improve the estimates of trajectory membership, particularly among participants with missing follow-up data. The post-deployment assessment time point was coded as 0, and each of the two subsequent follow-up assessments were coded as the number of months after the post-deployment assessment. The mean number of months between the first post-deployment assessment and each subsequent assessment were 2.38 (s.d. = 0.62) for the first follow-up, and 11.76 (s.d. = 1.74) for the final follow-up.

Associations between each PRS and trajectory membership were examined in multinomial logistic regression analyses with the *nnet* package (version 7.3-14; Venables & Ripley, 2002). Each model adjusted for sex, age, a total of eight PTE exposure variables from the pre-deployment and deployment phases, and within-ancestry variation using 10 principal components (Price et al., 2006). We also applied robust methods to deal with two potential sources of bias: (1) bias due to classification uncertainty in the trajectory models, and (2) selection bias arising from the exclusion of 546 participants who could not be included in the trajectory modeling because they did not have any PCL assessments in the post-deployment period. To take classification uncertainty into account, we used the probability-weighted regression approach (Clark & Muthén, 2009). In contrast to the ‘classify-analyze’ approach in which most-likely trajectory assignment is treated as an exact observed variable, the approach we took applied the participants' posterior probabilities of trajectory membership as weights in the regression model that examined PRS associations with trajectories (Clark & Muthén, 2009). To account for excluded participants, we derived inverse probability of response weights (Mansournia & Altman, 2016). Inverse probability of response weights were estimated using a stacked ensemble of machine learning algorithms (Gruber, Logan, Jarrin, Monge, & Hernan, 2015) with the *h2o* package (H2O.ai., 2020). In this approach, pre-deployment PCL and PTE exposure variables, six PRS, and 10 ancestral principal components were used to calculate cross-estimated probabilities of participation in at least one post-deployment assessment (i.e. the criterion for inclusion in trajectory analyses). Incorporating the inverse of these probabilities as weights in the regression analyses can reduce the bias of unweighted estimates based solely on the sample of included participants (Mansournia & Altman, 2016).

The posterior probabilities of trajectory membership and the inverse probability of response weights were multiplied and used as the weights in the regression analyses. For each multinomial regression analysis, PRS that were significant at the *p* < 0.0083 threshold (reflecting Bonferroni adjustment for six models) were probed by calculating adjusted odds ratios (AORs) across each pairwise trajectory combination (i.e. the PRS association adjusted for sex, age, ancestral components, and PTE exposure variables). Mean AORs with 95% confidence intervals (CIs) that did not overlap with 1 were considered significant at the *p* < 0.05 level.

## Results

### Posttraumatic stress symptom trajectories

Among participants included in the trajectory analyses, the average harmonized PCL score (range 0–100% of total) was 16.84% (s.d. = 18.79, median = 10%, IQR = 25; *n* = 3897) at post-deployment, 11.84% (s.d. = 16.31, median = 5.88%, IQR = 16.18; *n* = 3566) at the first follow-up, and 14.14% (s.d. = 19.39, median = 4.41%, IQR = 22.06; *n* = 3351) at the second follow-up. In the original scale, the mean score for the raw 5-item PCL (range 0–20) at post-deployment was 3.37 (s.d. = 3.76, median = 2, IQR = 5), and the mean scores for the raw 17-item PCL (range 0–68) were 8.05 (s.d. = 11.09, median = 4, IQR = 11) for the first follow-up and 9.62 (s.d. = 13.19, median = 3, IQR = 15) for the second follow-up. All models converged; we focused on the LGMMs because they had the best fit statistics. Models with increasing numbers of trajectories had higher log-likelihood, lower SSA-BIC, and significant LMR-LRT tests, which are indicative of improved fit (online Supplementary Table S4). However, the five-trajectory model had slightly lower entropy and added a trajectory that contained a small group of participants (2.66%) and was qualitatively similar to another trajectory in the model (online Supplementary Fig. S1). Moreover, the five-trajectory models showed variation across the modeling strategies, with one model identifying two increasing trajectories and two models identifying two decreasing trajectories. Finally, the four-trajectory LGMM contained the four trajectories that were most commonly observed in a comprehensive review of over 50 studies of trajectories after PTEs (Galatzer-Levy et al., 2018). Therefore, the four-trajectory LGMM was selected.

**Fig. 1.**
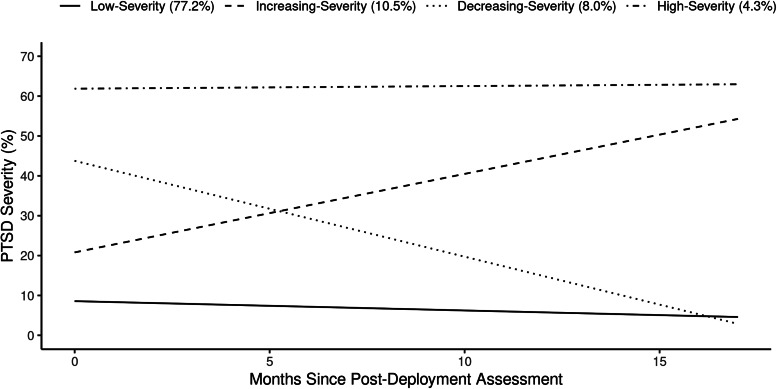
Plot of the posttraumatic stress symptom trajectories from the four-trajectory LGMM.

Table 1 provides parameter estimates from the four-trajectory LGMM and Fig. 1 provides an illustration of the trajectories. The model's entropy was 0.85 and the average of the posterior probabilities was 0.92; values above 0.80 and 0.70, respectively, are indicative of good classification of participants and separation of trajectories (Nylund-Gibson & Choi, 2018). The trajectories of posttraumatic stress symptoms followed patterns that have been previously described as resilient, recovering, delayed onset, and chronic (Galatzer-Levy et al., 2018). *Delayed onset* is used to describe a scenario in which posttraumatic stress symptoms begin to emerge sometime after the index trauma has passed. Since the assessments of posttraumatic stress symptoms in this study were not tied specifically to deployment stressors, it is also plausible that increasing levels of posttraumatic stress symptoms were related to new PTEs (as opposed to a delayed reaction to older PTEs). To assess this, we compared rates of new PTE exposures across trajectories using χ^2^ tests. Results showed that 63.6% of participants in the trajectory with increasing severity reported new exposure to PTEs in the follow-up window, which was significantly higher than the proportion reporting new PTEs in the low- (34.8%, *p* < 0.001) and decreasing-severity trajectories (50.6%, *p* < 0.001), but not significantly different than the high-severity trajectory (63.8%, *p* = 0.96). Therefore, rather than adopting the commonly used trajectory labels of resilient, recovering, delayed onset, and chronic, we applied the labels low-severity, decreasing-severity, increasing-severity, and high-severity.

**Table 1.** Parameter estimates from the final four-class LGMM

Trajectory label and description	Intercept (s.e.)	Linear slope (s.e.)	*n* (%)
Low-severity: low post-deployment severity, decreasing through follow-up	8.57 (0.22) *p* < 0.001	−0.23 (0.03) *p* < 0.001	3360 (77.2)
Decreasing-severity: moderate post-deployment severity, decreasing through follow-up	43.77 (1.23) *p* < 0.001	−2.40 (0.12) *p* < 0.001	350 (8.0)
Increasing-severity: low post-deployment severity, increasing through follow-up	20.80 (0.84) *p* < 0.001	1.97 (0.10) *p* < 0.001	456 (10.5)
High-severity: high post-deployment severity, stable through follow-up	61.87 (1.29) *p* < 0.001	0.07 (0.14) *p* = 0.64	188 (4.3)

*Note: n*, number of participants assigned to each trajectory based on the highest posterior probability of class membership.

The most common trajectory was low-severity (77.2%), characterized by having the lowest post-deployment severity which decreased through follow-up. The increasing-severity (10.5%) trajectory started with the second lowest post-deployment severity but increased through the follow-up to the moderate-high range of severity. Conversely, the decreasing-severity (8.0%) trajectory started in the moderate-high range and decreased through the follow-up to the level of the low-severity trajectory. Finally, the high-severity (4.3%) trajectory had the highest post-deployment severity, which remained stable through the follow-up. Online Supplementary Fig. S2 illustrates the individual observed values across these four trajectories.

### PRS associations with posttraumatic stress symptom trajectories

After controlling for age, sex, 10 ancestral components, and exposure to PTEs before or during their most recent deployment, the PRS for PTSD (*p* = 0.008) and MDD (*p* = 0.001) reached statistical significance in the multinomial logistic regression models; the remaining PRS were not significantly associated with trajectory membership (suicide attempt: *p* = 0.52; schizophrenia: *p* = 0.99; AUD: *p* = 0.51; neuroticism: *p* = 0.04). Results remained nearly identical after removing 52 participants (1.2%) that did not report any PTEs in their available assessments; therefore, we report statistics for the full sample.

**Fig. 2.**
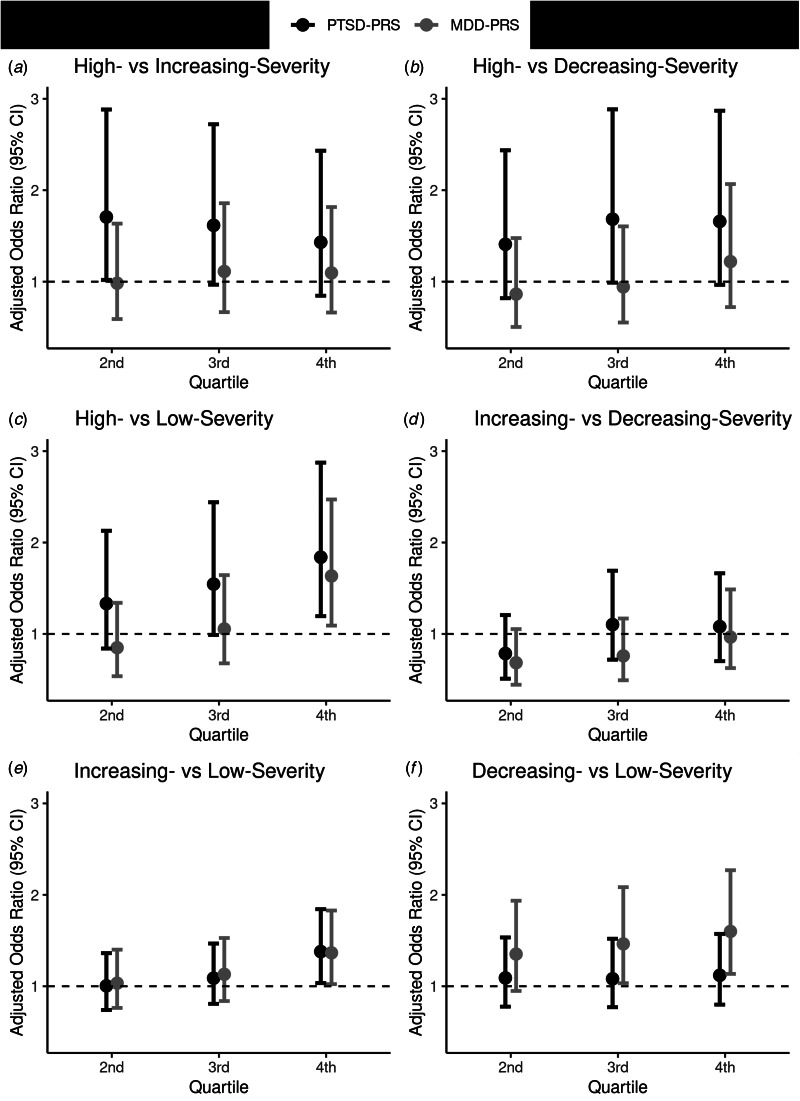
Plot of AORs for each PRS quartile relative to the first quartile.

Table 2 reports the AORs and 95% CIs for PTSD- and MDD-PRS across each of the trajectory pairs. PTSD-PRS was associated with significantly greater odds of membership in the high-severity *v.* low-severity trajectory, AOR = 1.23 (1.06–1.43), and the increasing-severity *v.* low-severity trajectory AOR = 1.12 (1.01–1.25). MDD-PRS was positively associated with significantly greater odds of membership in the high-severity *v.* low-severity trajectory, AOR = 1.18 (1.02–1.37), the increasing-severity *v.* low-severity trajectory, AOR = 1.16 (1.04–1.28), and the decreasing-severity *v.* low-severity trajectory, AOR = 1.16 (1.03–1.31). Figure 2 illustrates how the AORs vary at each PRS quartile relative to the lowest quartile for PTSD-PRS and MDD-PRS.

**Table 2.** AORs of PRS associations with posttraumatic stress symptom trajectories

	High-severity *v.* increasing-severity	High-severity *v.* decreasing-severity	High-severity *v.* low-severity	Increasing-severity *v.* decreasing-severity	Increasing-severity *v.* low-severity	Decreasing-severity *v.* low-severity
PTSD-PRS	1.09 (0.92–1.30)	1.13 (0.94–1.36)	**1.23 (1.06–1.43)**	1.04 (0.89–1.20)	**1.12 (1.01–1.25)**	1.08 (0.96–1.22)
MDD-PRS	1.02 (0.86–1.22)	1.02 (0.85–1.22)	**1.18 (1.02–1.37)**	0.99 (0.86–1.15)	**1.16 (1.04–1.29)**	**1.16 (1.03–1.31)**

*Note.* AORs greater than 1 reflect a positive association between PRS and membership in the first trajectory relative to the second. AORs with 95% CIs that do not cross 1 (in bold) are significant at the *p* < 0.05 level.

## Discussion

In this study of nearly 5000 US Army soldiers, higher PRS for PTSD and MDD were associated with more severe posttraumatic stress symptom trajectories after combat deployment to Afghanistan. High PTSD- and MDD-PRS were associated with increased odds of both chronically elevated posttraumatic stress symptoms and increasing severity of posttraumatic stress symptoms during the 9 months after return from deployment (relative to a trajectory characterized by consistently low posttraumatic stress symptoms). High MDD-PRS was further associated with increased odds of elevated posttraumatic stress symptoms in the initial reintegration phase (i.e. 1–3 months post-deployment), which resolved by 9 months post-deployment.

These results add to emerging evidence suggesting that PRS may help identify at-risk individuals (Choi et al., 2020; Joo et al., 2022; Schultebraucks, Choi, Galatzer-Levy, & Bonanno, 2021; Stein et al., 2021a), including those at risk of developing clinically significant posttraumatic stress symptoms following PTE exposure (Misganaw et al., 2019; Tamman et al., 2022; Waszczuk et al., 2020). Effect sizes in the current study were small and comparable to other studies, suggesting that further advancements are needed to increase the utility of PRS in predicting symptom patterns following major stressors (e.g. improved precision of PTSD- and MDD-PRS; combining PRS with information about other biological, behavioral, or environmental risk factors to better estimate PTSD risk; Schultebraucks et al., 2021). Of note, effect sizes reflect the unique contribution of individual PRS *over and above* demographic control variables and PTE exposure before and during the index deployment. Moreover, we used robust methods to account for the impact of missing data and set a conservative significance threshold to account for our examination of five PRS.

In addition to highlighting the potential value of PTSD- and MDD-PRS for predicting time course-related reactions following potential trauma exposure, this study provides information regarding the distribution of posttraumatic stress symptom trajectories of combat-deployed soldiers. More than three-quarters (77%) of soldiers followed a trajectory marked by low symptom severity, which suggests resilience, given the high levels of exposure to PTEs. The remainder of the sample exhibited either increasing severity (10%) that may reflect either delayed onset of clinically significant posttraumatic stress symptoms or elevations due to new PTE exposure, elevated posttraumatic stress symptoms during initial reintegration that resolved by approximately 9 months post-deployment (8%), or chronically elevated posttraumatic stress symptoms (4%). The distribution of class membership is remarkably consistent with that of deployed Army Reservists (Wang et al., 2018) and police responders to the World Trade Center attacks, which represents another group who pursued a high-risk occupation and were exposed to PTEs in the course of their work (Pietrzak et al., 2014).

To our knowledge, this study provides the first evidence of the potential utility of PRS in predicting increasing severity of posttraumatic stress symptoms (whether due to delayed symptom onset or symptom elevations due to new PTEs), which could be of unique value to prevention efforts. The associations of PTSD- and MDD-PRS with the increasing-severity trajectory imply that PRS could eventually be used to identify at-risk individuals who would not be easily detected at baseline through early symptom screening since they begin with relatively low posttraumatic stress symptom scores. Low-severity or asymptomatic individuals with genetic risk factors could be engaged in longer-term assessment protocols or provided with interventions to decrease the likelihood of onset of PTSD or associated problems (e.g. suicidal ideation; Wang et al., 2018) during the months following PTE exposure. In a military setting, risk mitigation efforts could be delivered to service members who report minimal symptoms during or shortly after returning from deployment (or after exposure to other PTEs), but whose PRS indicate elevated risk of PTSD or MDD. Similar applications could be envisioned with respect to other high-risk occupations (e.g. police officers, firefighters) or settings that provide early intervention to trauma survivors (e.g. emergency departments).

As noted earlier, some genetic susceptibility is shared across mental disorders and PRS can be predictive across diagnostic categories; thus, we examined several mental health-related PRS in relation to post-deployment posttraumatic stress symptoms. PRS for schizophrenia, AUD, suicide attempt, and neuroticism were not significantly associated with posttraumatic stress symptom trajectories, whereas both the PTSD-PRS and the MDD-PRS predicted more severe trajectories. Investigations of the latent structure of mental disorders consistently find that PTSD and MDD load together on a distress disorders subfactor of internalizing disorders (de Jonge et al., 2018), suggesting a high degree of shared vulnerability to these two disorders. Our results support the inference that risk factors that predispose individuals to depressive symptoms may also increase their risk of posttraumatic stress symptoms following trauma exposure. Indeed, MDD-PRS was a somewhat stronger predictor of posttraumatic stress symptom trajectories than PTSD-PRS, differentiating all three ‘symptomatic’ trajectories from the low-severity trajectory. The version of the MDD-PRS used here comes from a much larger case sample than the version of the PTSD-PRS used here, which may have contributed to its seemingly greater predictive power. Alternatively, there may be specific aspects of MDD vulnerability that relate to changes in posttraumatic stress symptoms across time. Further exploration of MDD-related posttraumatic outcomes may be warranted.

### Limitations

Several study limitations must be considered in interpreting the results. First, due to limited availability of reference GWAS data in other populations, we were only able to study the associations of PRS with posttraumatic stress symptom trajectories among soldiers of European ancestry. The current findings may not generalize to soldiers of non-European ancestry, and it will be important for future studies to evaluate the predictive value of PRS among individuals of other ancestral backgrounds. Second, the assessment of posttraumatic stress symptoms was based on self-report, a modality that is vulnerable to recall and response biases. Future studies should examine whether PRS also predict clinician ratings of posttraumatic stress symptom severity or PTSD diagnosis. Third, the pre-deployment and 1-month post-deployment surveys contained abbreviated versions of the PCL-C that did not provide full coverage of the PTSD construct as defined in standard diagnostic manuals. Fourth, the availability of only three time-points after deployment precluded modeling non-linear trajectories. Fifth, our trajectory analyses span approximately a 9-month period after service members returned from deployment and should not be interpreted beyond that time window. Sixth, simulation studies have shown that probability-weighted regression can underestimate the standard errors of the associations between variables of interest and trajectory membership relative to an approach in which the variables are used as covariates in the trajectory modeling process (Clark & Muthén, 2009). Finally, following presentations of PTE checklists, the assessment of posttraumatic stress symptoms in the PPDS was anchored to ‘any stressful experience’. It is possible that some respondents provided ratings of symptoms that occurred in response to experiences that would not be clinically categorized as traumatic events.

## Conclusions

The current study found that higher polygenic risk for PTSD or MDD is associated with more severe posttraumatic stress symptom trajectories following combat deployment. More research is needed to replicate the current results and to achieve better precision in characterizing an individual's genetic risks for adverse posttraumatic outcomes. With further advancement, PRS or other measures of genetic risk could be used in conjunction with other screening tools, to improve the accuracy of PTSD risk assessment and enable more precise targeting of prevention programs. This could enhance the cost-effectiveness of treatment and prevention efforts, permitting higher intensity monitoring and support for the most vulnerable individuals.

## Supplementary material

For supplementary material accompanying this paper visit https://doi.org/10.1017/S0033291723000211. click here to view supplementary material
