# Supplementary material for: Associations of polygenic risk scores with posttraumatic stress symptom trajectories following combat deployment
Source: Psychol Med. 2023 Mar 6;53(14):6733–42. doi: 10.1017/S0033291723000211 (PMC10480347; doi:10.1017/S0033291723000211)
Supplement: Supplementary file 1 [file S0033291723000211sup001.pdf]

Supplementary Material for *Associations of polygenic risk scores with posttraumatic stress symptom trajectories following combat deployment*

Table of Contents

Supplementary Table 1. Summary data sources for polygenic risk scores

Supplementary Table 2. Correlations between scores calculated using the full and reduced versions of the PCL at the follow-ups

Supplementary Table 3. Guidelines for reporting on latent trajectory studies (checklist)

Supplementary Table 4. Model fit indices of the estimated models

Supplementary Figure 1. PTSD trajectories from each of the estimated models

Supplementary Figure 2. Individual observed trajectories for each LGMM trajectory

Appendix 1. Analysis code and output of the LGMM models (random intercepts and slopes)

Appendix 2. Analysis code and output of the LGMM models (random intercepts, fixed slopes)

Appendix 3. Analysis code and output of the LCGA models (fixed intercepts and slopes)

Appendix 4. PRS associations with trajectories after controlling for additional variables suggested by reviewers

Supplementary Table 1. Data sources for polygenic risk scores

Phenotype	Summary Statistics Description & Sample Size	Summary Statistics Source
PTSD (Stein et al., 2021)	Continuous severity based on PCL total scores. N = 186689	https://www.ncbi.nlm.nih.gov/projects/gap/cgi-bin/study.cgi?study_id=phs001672.v1.p1.
MDD (Howard et al., 2019)	Diagnosis based on clinician diagnosis, chart review, self-reported symptoms. N = 500199	https://datashare.ed.ac.uk/handle/10283/3203
Suicide attempt (Mullins et al., 2022)	Incidence based on structured clinical interviews, self-report questionnaires, and hospital records or ICD codes. N = 538436 (STARRS sample removed)	https://doi.org/10.1016/j.biopsych.2021.05.029
Schizophrenia (Ripke et al., 2020)	Diagnosis based on clinician and/or research assessment. N = 306011	https://figshare.com/articles/dataset/scz2021/14672178
Alcohol Use Disorder (Walters et al., 2018)	Diagnosis based on clinician ratings or semi-structured interviews. N = 38686	https://figshare.com/articles/dataset/sud2018-alc/14672187
Neuroticism (Nagel et al., 2018)	Continuous measure based on 12 items of the Eysenck Personality Questionnaire Revised Short Form. N = 390278	https://ctg.cncr.nl/software/summary_statistics

Supplementary Table 2. Correlations between scores calculated using the full and reduced versions of the PCL at the follow-ups

	6-item score and 17-item score<br>*R* [95% CI], *p-value*	5-item score and 17-item score<br>*R* [95% CI], *p-value*
Follow-up 1	.96 [.96, .96], $p < .001$	.95 [.95, .96], $p < .001$
Follow-up 2	.95 [.95, .96], $p < .001$	.95 [.95, .96], $p < .001$

Note. The 6-item score reflects the sum of the six PCL items collected at pre-deployment, the 5-item score reflects the sum of the five PCL items collected at post-deployment, and the 17-item score reflects the sum of the 17 PCL items (i.e., 17 DSM-IV PTSD symptoms).

Supplementary Table 3. Guidelines for reporting on latent trajectory studies (checklist)

Checklist Item	*Section Where Item is Reported*
**1. Is the metric of time used in the statistical model reported?**<br>*Yes: "The post-deployment assessment timepoint was coded as 0, and each of the two subsequent follow-up assessments were coded as the number of months after the post-deployment assessment."*	Method\Data analysis
**2. Is information presented about the mean and variance of time within a wave?**<br>*Yes: "The mean number of months between the first post-deployment assessment and each subsequent assessment were 2.38 (SD = 0.62) for the first follow-up, and 11.76 (SD = 1.74) for the final follow-up."*	Method\Data analysis
**3a. Is the missing data mechanism reported?**<br>*Yes (missing at random): "Trajectory analyses were conducted in R (R Core Team, 2022) with the lcmm package (version 1.9. 4;* Proust-Lima *et al.*, 2017*), which uses maximum likelihood estimation to include participants with data assumed to be missing at random."*	Method\Data analysis
**3b. Is a description provided of what variables are related to attrition/missing data?**<br>*Yes: "A non-monotonic missing data pattern was observed such that only a minority of participants (3.5%) completed the post-deployment assessment but did not return for any follow-ups. Pre-deployment posttraumatic stress symptom severity was significantly higher among participants with missing data at post-deployment (M difference = 3.50, p < .001) and at the first follow-up (M difference = 3.03, p < .001), but not the last follow-up (M difference = -0.48, p = .37)."*	Method\Data analysis
**3c. Is a description provided of how missing data in the analyses were dealt with?**<br>*Yes (maximum likelihood estimation): "Trajectory analyses were conducted in R (R Core Team, 2022) with the lcmm package (version 1.9.4;* Proust-Lima *et al.*, 2017*), which uses maximum likelihood estimation to include participants with data assumed to be missing at random."*	Method\Data analysis
**4. Is information about the distribution of the observed variables included?**<br>*Yes: "Among participants included in the trajectory analyses, the average harmonized PCL score (range 0-100% of total) was 16.84% (SD = 18.79, median = 10%, IQR = 25; n = 3897) at post-deployment, 11.84% (SD = 16.31, median = 5.88%, IQR = 16.18; n = 3566) at the first follow-up, and 14.14% (SD = 19.39, median = 4.41%, IQR = 22.06; n = 3351) at the second follow-up."*	Results\Sample Characteristics

**5. Is the software mentioned?**<br>*Yes: "Trajectory analyses were conducted using the open source software R (R Core Team, 2022) with the lcmm package (version 1.9. 4; Proust-Lima et al., 2017..."*	Method\Analysis
**6a. Are alternative specifications of within-class heterogeneity considered (e.g., LGCA vs. LGMM) and clearly documented? If not, was sufficient justification provided as to eliminate certain specifications from consideration?**<br>*Yes: "We examined modeling approaches that varied in their specifications of within-class heterogeneity: latent growth mixture models (LGMM; within-trajectory variation in the intercepts and/or slopes is allowed) and latent class growth analysis (LCGA; within-trajectory variance of the intercepts and slopes are fixed to zero)"*	Method\Data analysis
**6b. Are alternative specifications of the between-class differences in variance–covariance matrix structure considered and clearly documented? If not, was sufficient justification provided as to eliminate certain specifications from consideration?**<br>*Justification provided: "The variance-covariance matrix of the random effects was constrained to be equal across trajectories to facilitate model convergence."*	Method\Data analysis
**7. Are alternative shape/functional forms of the trajectories described?**<br>*Yes: "We selected the post-deployment assessment as the starting point of our trajectory analyses (described in Data Analysis). This allowed us to model the course of posttraumatic stress symptom trajectories after exposure to potentially traumatic experiences during deployment. Additionally, length of deployment differed across the three BCTs, making the time from pre- to post-deployment assessments highly variable, which would have limited the interpretability of the trajectories."*	Method\Data analysis
**8. If covariates have been used, can analyses still be replicated?**<br>*Yes: "Associations between each PRS and trajectory membership were examined in multinomial logistic regression analyses with the nnet package (version 7.3-14; Venables and Ripley, 2002)..."*	Method\Data analysis
**9. Is information reported about the number of random start values and final iterations included?**<br>*Yes: "Random starting values for the multi-trajectory models used information from the single-trajectory model; values were generated using grid-search with 100 repetitions and a maximum of 100 iterations in the optimization algorithm. Analysis outputs with precise information about the number of random start values and final iterations included are provided in the Supplementary Material."*	Method\Data analysis & Supplementary Material

**10. Are the model comparison (and selection) tools described from a statistical perspective?**<br>*Yes: "We compared models with one to five trajectories and selected a final model based on sample size adjusted Bayesian information criteria (lower is better), entropy (reflects classification accuracy; values closer to 1 are better), and the Lo-Mendell-Rubin likelihood ratio test (significant value indicates better fit). Parsimonious models were favored over models that improved fit by adding an insufficiently distinct trajectory that included a low proportion of participants."*	Method\Data analysis
**11. Are the total number of fitted models reported, including a one-class solution?**<br>*Yes, Supplementary Table S2 shows the fit indices of the LGMM results with 1-5 trajectories.*	Supplementary Table 2
**12. Are the number of cases per class reported for each model (absolute sample size, or proportion)?**<br>*Yes, reported in Table 1.*	Table 1
**13. If classification of cases in a trajectory is the goal, is entropy reported?**<br>*Yes, Supplementary Table 2 reports entropy of the LGMM results with 1-5 trajectories.*	Supplementary Table 2
**14a. Is a plot included with the estimated mean trajectories of the final solution?**<br>*Yes: Figure 1.*	Figure 1
**14b. Are plots included with the estimated mean trajectories for each model?**<br>*Yes: Supplementary Figure 2.*	Supplementary Figure 2
**14c. Is a plot included of the combination of estimated means of the final model and the observed individual trajectories split out for each latent class?**<br>*Yes: Supplementary Figure 1.*	Supplementary Figure 1
**15. Are characteristics of the final class solution numerically described (i.e., means, SD/SE, n, CI, etc.)?**<br>*Reported in Table 1.*	Table 1
**16. Are the syntax files available (either in the appendix, supplementary materials, or from the authors)?**<br>*Provided in Appendix 1-3 of the Supplementary Materials.*	Supplementary Materials

Supplementary Table 4. Model fit indices of the different models.

Model	Log Likelihood	AIC	BIC	SSA-BIC	Entropy	% in Smallest Trajectory	LMR-LRT (*p*-value)
LGMM							
1 trajectory	-45504.87	91021.75	91060.02	91040.96	1	100	NA
2 trajectories	-44550.01	89120.02	89183.81	89152.03	0.90	13.50	1836.7 (<.001)
3 trajectories	-44243.42	88514.84	88604.15	88559.66	0.85	6.64	589.7 (<.001)
4 trajectories	-43892.01	87820.01	87934.83	87877.64	0.86	4.32	675.9 (<.001)
5 trajectories	-43756.79	87557.58	87697.91	87628.01	0.85	2.66	260.1 (<.001)
LGMM with fixed slopes							
1 trajectory	-45585.63	91179.26	91204.78	91192.07	1	100	NA
2 trajectories	-44640.54	89297.08	89348.11	89322.69	0.90	14.10	1817.9 (<.001)
3 trajectories	-44193.72	88411.44	88487.99	88449.86	0.89	7.56	859.4 (<.001)
4 trajectories	-43897.56	87827.13	87929.19	87878.35	0.86	4.25	569.7 (<.001)
5 trajectories	-43772.34	87584.68	87712.25	87648.7	0.85	2.07	240.9 (<.001)
LCGA							
1 trajectory	-46794.13	93594.25	93613.39	93603.85	1	100	NA
2 trajectories	-44745.41	89504.83	89549.48	89527.24	0.91	16.72	3940.7 (<.001)
3 trajectories	-44321.82	88665.64	88735.8	88700.85	0.87	6.01	814.8 (<.001)
4 trajectories	-43929.83	87889.65	87985.34	87937.67	0.86	5.26	754.0 (<.001)
5 trajectories	-43772.34	87582.68	87703.87	87643.5	0.85	2.07	302.9 (<.001)

Note. SSA-BIC = Sample Size Adjusted Bayesian Information Criteria; LMR-LRT = Lo-Mendell-Rubin Likelihood Ratio Test for all of the estimated models.

Supplementary Figure 1. PTSD trajectories from each of the estimated models

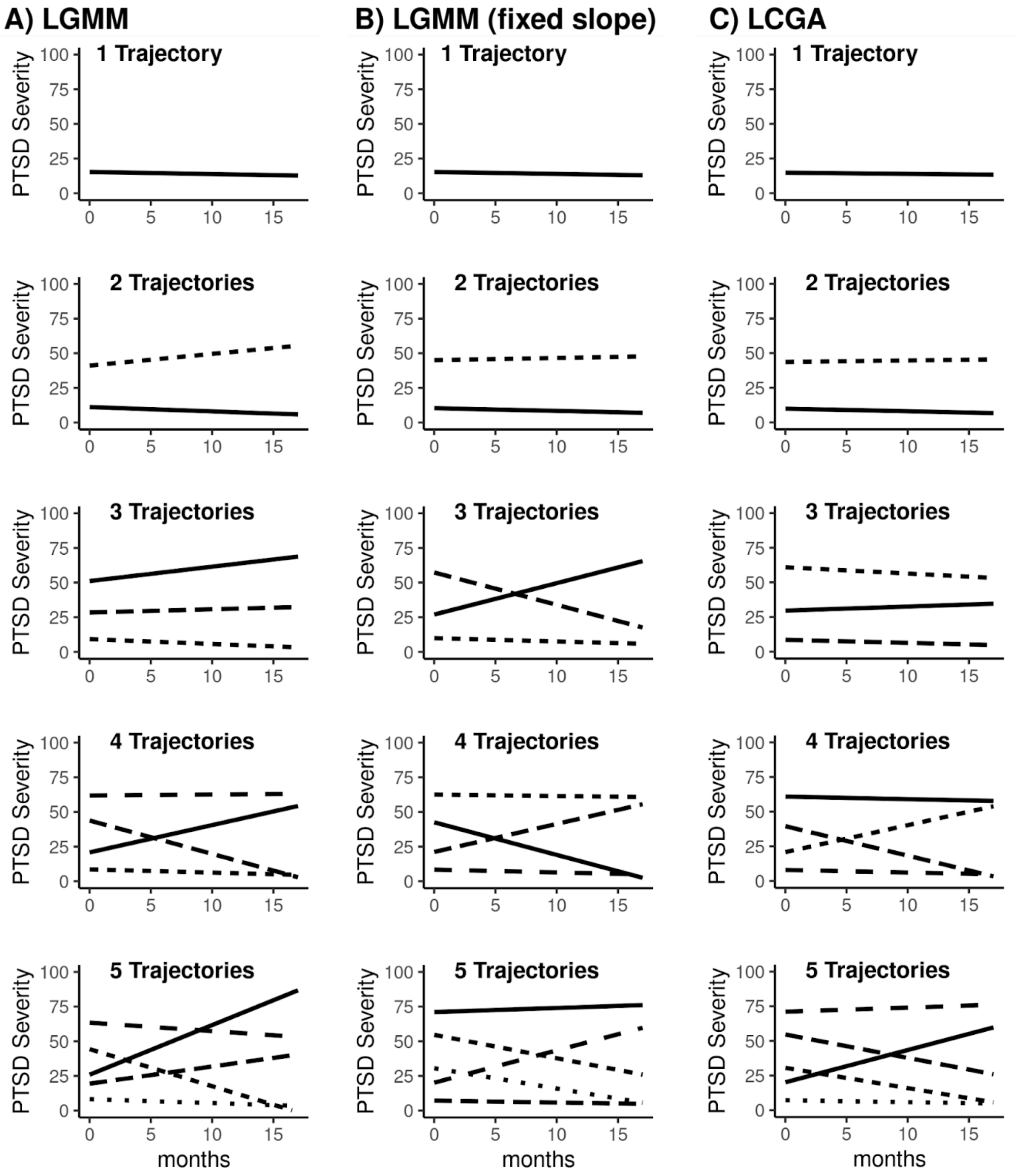

Supplementary Figure 2. Individual observed trajectories for each LGMM trajectory.

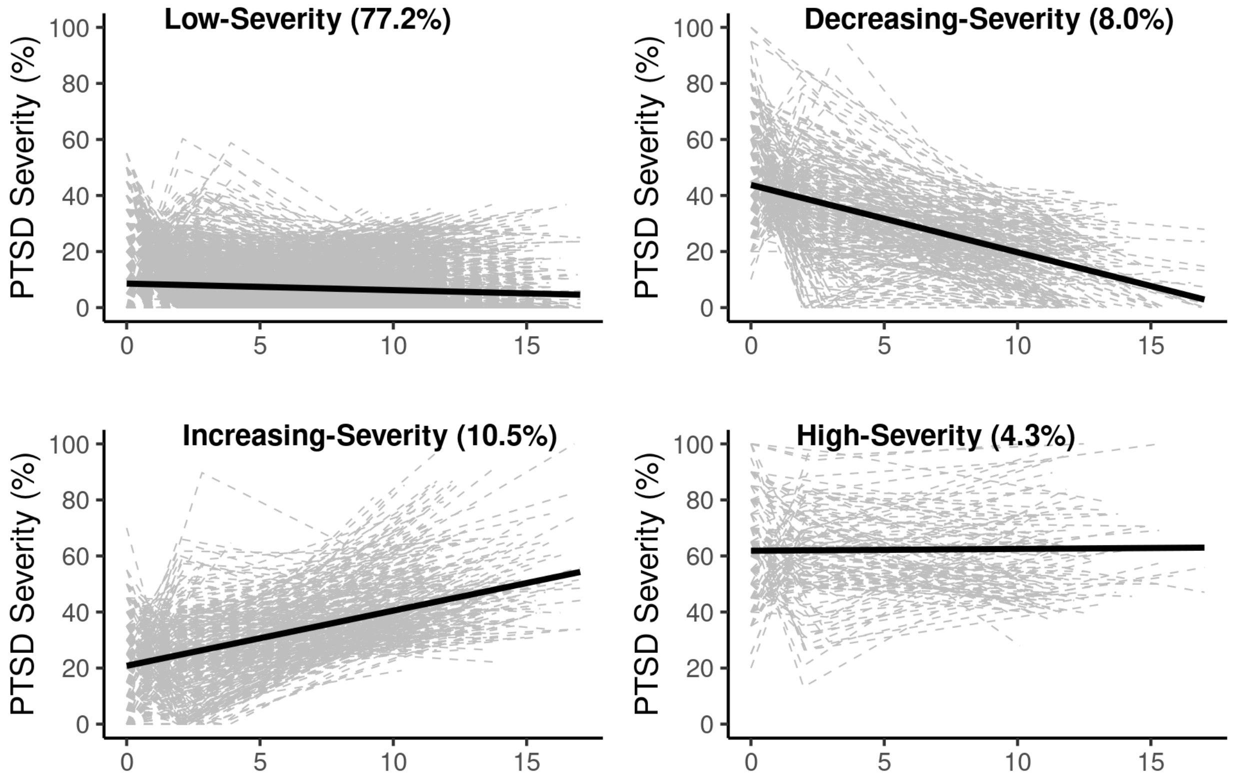

Appendix 1. Analysis code and output of the LGMM models (random intercepts and slopes)

```
#Syntax
library(lcmm)
#1 trajectory
lgmm1 <- hlme(pcl ~ months,  subject = "id", ng = 1, data = df,
verbose = F, returndata = TRUE, random = ~1 + months)
summary(lgmm1)
saveRDS(lgmm1, "lgmm1.prs.rds")
#2 trajectories
lgmm2 <- gridsearch(rep = 100, maxiter = 100, minit = lgmm1, cl = 16,
                    hlme(pcl ~ months,  subject = "id", ng = 2, data =
df, mixture = ~1 + months, verbose = F, returndata = TRUE, classmb =
~pcl.bl, random = ~1 + months))
summary(lgmm2)
saveRDS(lgmm2, "lgmm2.prs.rds")
#3 trajectories
lgmm3 <- gridsearch(rep = 100, maxiter = 100, minit = lgmm1, cl = 16,
                    hlme(pcl ~ months,  subject = "id", ng = 3, data =
df, mixture = ~1 + months, verbose = F, returndata = TRUE, classmb =
~pcl.bl, random = ~1 + months))
summary(lgmm3)
saveRDS(lgmm3, "lgmm3.prs.rds")
#4 trajectories
lgmm4 <- gridsearch(rep = 100, maxiter = 100, minit = lgmm1, cl = 16,
                    hlme(pcl ~ months,  subject = "id", ng = 4, data =
df, mixture = ~1 + months, verbose = F, returndata = TRUE, classmb =
~pcl.bl, random = ~1 + months))
summary(lgmm4)
saveRDS(lgmm4, "lgmm4.prs.rds")
#5 trajectories
lgmm5 <- gridsearch(rep = 100, maxiter = 100, minit = lgmm1, cl = 16,
                    hlme(pcl ~ months,  subject = "id", ng = 5, data =
df, mixture = ~1 + months, verbose = F, returndata = TRUE, classmb =
~pcl.bl, random = ~1 + months))
summary(lgmm5)
saveRDS(lgmm5, "lgmm5.prs.rds")
sessionInfo()

#Output
Heterogenous linear mixed model
     fitted by maximum likelihood method

hlme(fixed = pcl ~ months, random = ~1 + months, subject = "id",
    ng = 1, data = df, verbose = F, returndata = TRUE)

Statistical Model:
     Dataset: df
     Number of subjects: 4355
     Number of observations: 10815
     Number of observations deleted: 3885
```

```
     Number of latent classes: 1
     Number of parameters: 6

Iteration process:
     Convergence criteria satisfied
     Number of iterations:  15
     Convergence criteria: parameters= 2.8e-11
                         : likelihood= 1.5e-09
                         : second derivatives= 7.1e-14

Goodness-of-fit statistics:
     maximum log-likelihood: -45504.87
     AIC: 91021.75
     BIC: 91060.02


Maximum Likelihood Estimates:

Fixed effects in the longitudinal model:

               coef       Se    Wald p-value
intercept  15.30635 0.26667  57.397 0.00000
months     -0.15123 0.02739  -5.521 0.00000


Variance-covariance matrix of the random-effects:
          intercept  months
intercept 208.44789
months     -3.12414 0.83152

                              coef      Se
Residual standard error:  11.13530 0.13596

Be patient, grid search is running ...
Search completed, performing final estimation
Heterogenous linear mixed model
     fitted by maximum likelihood method

hlme(fixed = pcl ~ months, mixture = ~1 + months, random = ~1 +
    months, subject = "id", classmb = ~pcl.bl, ng = 2, data = df,
    verbose = F, returndata = TRUE)

Statistical Model:
     Dataset: df
     Number of subjects: 4354
     Number of observations: 10814
     Number of observations deleted: 3886
     Number of latent classes: 2
     Number of parameters: 10

Iteration process:
```

```
     Convergence criteria satisfied
     Number of iterations:  1
     Convergence criteria: parameters= 2.4e-13
                         : likelihood= 4.4e-11
                         : second derivatives= 3.5e-13

Goodness-of-fit statistics:
     maximum log-likelihood: -44550.01
     AIC: 89120.02
     BIC: 89183.81

Maximum Likelihood Estimates:

Fixed effects in the class-membership model:
(the class of reference is the last class)

                      coef      Se    Wald p-value
intercept class1   2.43690 0.07077  34.435 0.00000
pcl.bl class1     -0.05377 0.00301 -17.862 0.00000

Fixed effects in the longitudinal model:

                      coef      Se    Wald p-value
intercept class1  11.16041 0.25299  44.115 0.00000
intercept class2  41.07676 0.88145  46.601 0.00000
months class1     -0.31136 0.02855 -10.905 0.00000
months class2      0.84421 0.09490   8.896 0.00000

Variance-covariance matrix of the random-effects:
          intercept months
intercept 103.38601
months     -6.58259 0.5267

                              coef      Se
Residual standard error:  11.09340 0.13379

Be patient, grid search is running ...
Search completed, performing final estimation
Heterogenous linear mixed model
     fitted by maximum likelihood method

hlme(fixed = pcl ~ months, mixture = ~1 + months, random = ~1 +
    months, subject = "id", classmb = ~pcl.bl, ng = 3, data = df,
    verbose = F, returndata = TRUE)

Statistical Model:
     Dataset: df
     Number of subjects: 4354
     Number of observations: 10814
     Number of observations deleted: 3886
     Number of latent classes: 3
```

```
     Number of parameters: 14

Iteration process:
     Convergence criteria satisfied
     Number of iterations:  7
     Convergence criteria: parameters= 4.2e-13
                         : likelihood= 3.3e-10
                         : second derivatives= 3.6e-10

Goodness-of-fit statistics:
     maximum log-likelihood: -44243.42
     AIC: 88514.84
     BIC: 88604.15

Maximum Likelihood Estimates:

Fixed effects in the class-membership model:
(the class of reference is the last class)

                     coef      Se    Wald p-value
intercept class1 -1.27708 0.12128 -10.530 0.00000
intercept class2  1.96632 0.06885  28.560 0.00000
pcl.bl class1     0.01612 0.00334   4.827 0.00000
pcl.bl class2    -0.05725 0.00385 -14.884 0.00000

Fixed effects in the longitudinal model:

                     coef      Se    Wald p-value
intercept class1 51.09980 1.23129  41.501 0.00000
intercept class2  9.20397 0.26353  34.926 0.00000
intercept class3 28.40806 0.78870  36.019 0.00000
months class1     1.03706 0.13486   7.690 0.00000
months class2    -0.34982 0.03186 -10.980 0.00000
months class3     0.23262 0.08764   2.654 0.00795

Variance-covariance matrix of the random-effects:
          intercept  months
intercept  75.99035
months     -7.51830 0.74384

                             coef      Se
Residual standard error: 10.52933 0.12642

Be patient, grid search is running ...
Search completed, performing final estimation
Heterogenous linear mixed model
     fitted by maximum likelihood method

hlme(fixed = pcl ~ months, mixture = ~1 + months, random = ~1 +
    months, subject = "id", classmb = ~pcl.bl, ng = 4, data = df,
    verbose = F, returndata = TRUE)
```

```
 Statistical Model:
     Dataset: df
     Number of subjects: 4354
     Number of observations: 10814
     Number of observations deleted: 3886
     Number of latent classes: 4
     Number of parameters: 18

Iteration process:
     Convergence criteria satisfied
     Number of iterations:  1
     Convergence criteria: parameters= 5.1e-10
                         : likelihood= 2.9e-09
                         : second derivatives= 4.1e-10

Goodness-of-fit statistics:
     maximum log-likelihood: -43892.01
     AIC: 87820.01
     BIC: 87934.83

Maximum Likelihood Estimates:

Fixed effects in the class-membership model:
(the class of reference is the last class)

                      coef      Se    Wald p-value
intercept class1  1.43755 0.15816   9.089 0.00000
intercept class2  3.80563 0.14248  26.711 0.00000
intercept class3  1.10883 0.16564   6.694 0.00000
pcl.bl class1    -0.02297 0.00411  -5.586 0.00000
pcl.bl class2    -0.07965 0.00454 -17.548 0.00000
pcl.bl class3    -0.01937 0.00430  -4.505 0.00001

Fixed effects in the longitudinal model:

                      coef      Se    Wald p-value
intercept class1 20.80435 0.83530  24.906 0.00000
intercept class2  8.56505 0.22412  38.216 0.00000
intercept class3 43.76547 1.22642  35.685 0.00000
intercept class4 61.86607 1.29297  47.848 0.00000
months class1     1.96858 0.09538  20.640 0.00000
months class2    -0.23333 0.02769  -8.427 0.00000
months class3    -2.40431 0.11943 -20.132 0.00000
months class4     0.06508 0.14336   0.454 0.64987

Variance-covariance matrix of the random-effects:
          intercept  months
intercept  22.84574
months     -0.77236 0.02611

                              coef      Se
```

```
Residual standard error: 10.55853 0.10292

Be patient, grid search is running ...
Search completed, performing final estimation
Heterogenous linear mixed model
     fitted by maximum likelihood method

hlme(fixed = pcl ~ months, mixture = ~1 + months, random = ~1 +
    months, subject = "id", classmb = ~pcl.bl, ng = 5, data = df,
    verbose = F, returndata = TRUE)

Statistical Model:
     Dataset: df
     Number of subjects: 4354
     Number of observations: 10814
     Number of observations deleted: 3886
     Number of latent classes: 5
     Number of parameters: 22

Iteration process:
     Convergence criteria satisfied
     Number of iterations:  4
     Convergence criteria: parameters= 3.4e-10
                         : likelihood= 1.3e-06
                         : second derivatives= 5e-07

Goodness-of-fit statistics:
     maximum log-likelihood: -43756.79
     AIC: 87557.58
     BIC: 87697.91

Maximum Likelihood Estimates:

Fixed effects in the class-membership model:
(the class of reference is the last class)

                     coef      Se    Wald p-value
intercept class1 -3.65972 0.19682 -18.594 0.00000
intercept class2 -2.78195 0.11091 -25.084 0.00000
intercept class3 -2.19165 0.08185 -26.776 0.00000
intercept class4 -3.83536 0.15103 -25.395 0.00000
pcl.bl class1     0.06506 0.00615  10.588 0.00000
pcl.bl class2     0.06279 0.00477  13.161 0.00000
pcl.bl class3     0.05881 0.00460  12.774 0.00000
pcl.bl class4     0.08518 0.00504  16.895 0.00000

Fixed effects in the longitudinal model:

                     coef      Se    Wald p-value
intercept class1 25.87088 2.03726  12.699 0.00000
intercept class2 44.31612 1.21380  36.510 0.00000
```

```
intercept class3 19.41817 0.83901  23.144 0.00000
intercept class4 63.39375 1.41826  44.698 0.00000
intercept class5  8.23760 0.22381  36.807 0.00000
months class1     3.58053 0.29505  12.135 0.00000
months class2    -2.67192 0.11874 -22.503 0.00000
months class3     1.23878 0.09023  13.729 0.00000
months class4    -0.60382 0.17385  -3.473 0.00051
months class5    -0.28394 0.02658 -10.681 0.00000

Variance-covariance matrix of the random-effects:
          intercept months
intercept  26.62199
months     -1.55051 0.0903

                             coef      Se
Residual standard error: 10.05975 0.10128

R version 4.1.0 (2021-05-18)
Platform: x86_64-pc-linux-gnu (64-bit)
Running under: CentOS Linux 7 (Core)

Matrix products: default
BLAS:   /sw/arcts/centos7/stacks/gcc/8.2.0/R/4.1.0/lib64/R/lib/libRbla
s.so
LAPACK:
/sw/arcts/centos7/stacks/gcc/8.2.0/R/4.1.0/lib64/R/lib/libRlapack.so

Random number generation:
 RNG:     L'Ecuyer-CMRG
 Normal:  Inversion
 Sample:  Rejection

locale:
 [1] LC_CTYPE=en_US.UTF-8       LC_NUMERIC=C
 [3] LC_TIME=en_US.UTF-8        LC_COLLATE=en_US.UTF-8
 [5] LC_MONETARY=en_US.UTF-8    LC_MESSAGES=en_US.UTF-8
 [7] LC_PAPER=en_US.UTF-8       LC_NAME=C
 [9] LC_ADDRESS=C               LC_TELEPHONE=C
[11] LC_MEASUREMENT=en_US.UTF-8 LC_IDENTIFICATION=C

attached base packages:
[1] parallel  stats     graphics  grDevices
utils     datasets  methods
[8] base

other attached packages:
[1] lcmm_1.9.3      mvtnorm_1.1-3   survival_3.2-11

loaded via a namespace (and not attached):
[1] compiler_4.1.0  Matrix_1.3-3    splines_4.1.0   grid_4.1.0
[5] lattice_0.20-44
```

Appendix 2. Analysis code and output of the LGMM models (random intercepts, fixed slopes)

```
#Syntax
library(lcmm)
#1 trajectory
lgmmfs1 <- hlme(pcl ~ months,  subject = "id", ng = 1, data = df,
verbose = F, returndata = TRUE, random = ~1)
summary(lgmmfs1)
saveRDS(lgmmfs1, "lgmmfs1.prs.rds")

#2 trajectories
lgmmfs2 <- gridsearch(rep = 100, maxiter = 100, minit = lgmmfs1, cl =
16, hlme(pcl ~ months,  subject = "id", ng = 2, data = df, mixture =
~1 + months, verbose = F, returndata = TRUE, classmb = ~pcl.bl, random
= ~1))
summary(lgmmfs2)
saveRDS(lgmmfs2, "lgmmfs2.prs.rds")

#3 trajectories
lgmmfs3 <- gridsearch(rep = 100, maxiter = 100, minit = lgmmfs1, cl =
16, hlme(pcl ~ months,  subject = "id", ng = 3, data = df, mixture =
~1 + months, verbose = F, returndata = TRUE, classmb = ~pcl.bl, random
= ~1))
summary(lgmmfs3)
saveRDS(lgmmfs3, "lgmmfs3.prs.rds")

#4 trajectories
lgmmfs4 <- gridsearch(rep = 100, maxiter = 100, minit = lgmmfs1, cl =
16, hlme(pcl ~ months,  subject = "id", ng = 4, data = df, mixture =
~1 + months, verbose = F, returndata = TRUE, classmb = ~pcl.bl, random
= ~1))
summary(lgmmfs4)
saveRDS(lgmmfs4, "lgmmfs4.prs.rds")

#5 trajectories
lgmmfs5 <- gridsearch(rep = 100, maxiter = 100, minit = lgmmfs1, cl =
16, hlme(pcl ~ months,  subject = "id", ng = 5, data = df, mixture =
~1 + months, verbose = F, returndata = TRUE, classmb = ~pcl.bl, random
= ~1))
summary(lgmmfs5)
saveRDS(lgmmfs5, "lgmmfs5.prs.rds")
sessionInfo()

#Output
Heterogenous linear mixed model
     fitted by maximum likelihood method

hlme(fixed = pcl ~ months, random = ~1, subject = "id", ng = 1,
    data = df, verbose = F, returndata = TRUE)

Statistical Model:
     Dataset: df
```

```
     Number of subjects: 4355
     Number of observations: 10815
     Number of observations deleted: 3885
     Number of latent classes: 1
     Number of parameters: 4

Iteration process:
     Convergence criteria satisfied
     Number of iterations:  15
     Convergence criteria: parameters= 1.2e-06
                         : likelihood= 2.9e-05
                         : second derivatives= 9.3e-13

Goodness-of-fit statistics:
     maximum log-likelihood: -45585.63
     AIC: 91179.26
     BIC: 91204.78

Maximum Likelihood Estimates:

Fixed effects in the longitudinal model:

               coef      Se    Wald p-value
intercept  15.28150 0.26600  57.450 0.00000
months     -0.13672 0.02460  -5.557 0.00000

Variance-covariance matrix of the random-effects:
          intercept
intercept  188.6323

                              coef      Se
Residual standard error:  12.44031 0.11078

Be patient, grid search is running ...
Search completed, performing final estimation
Heterogenous linear mixed model
     fitted by maximum likelihood method

hlme(fixed = pcl ~ months, mixture = ~1 + months, random = ~1,
    subject = "id", classmb = ~pcl.bl, ng = 2, data = df, verbose = F,
    returndata = TRUE)

Statistical Model:
     Dataset: df
     Number of subjects: 4354
     Number of observations: 10814
     Number of observations deleted: 3886
     Number of latent classes: 2
     Number of parameters: 8

Iteration process:
```

```
     Convergence criteria satisfied
     Number of iterations:  1
     Convergence criteria: parameters= 1.5e-12
                         : likelihood= 1.4e-10
                         : second derivatives= 2.8e-12

Goodness-of-fit statistics:
     maximum log-likelihood: -44640.54
     AIC: 89297.08
     BIC: 89348.11

Maximum Likelihood Estimates:

Fixed effects in the class-membership model:
(the class of reference is the last class)

                     coef      Se    Wald p-value
intercept class1  2.43165 0.07182  33.858 0.00000
pcl.bl class1    -0.05644 0.00311 -18.145 0.00000

Fixed effects in the longitudinal model:

                     coef      Se    Wald p-value
intercept class1 10.34588 0.21786  47.490 0.00000
intercept class2 45.01607 0.72126  62.413 0.00000
months class1    -0.19437 0.02717  -7.155 0.00000
months class2     0.15521 0.08213   1.890 0.05878


Variance-covariance matrix of the random-effects:
          intercept
intercept  31.97744

                             coef      Se
Residual standard error: 12.31836 0.10784

Be patient, grid search is running ...
Search completed, performing final estimation
Heterogenous linear mixed model
     fitted by maximum likelihood method

hlme(fixed = pcl ~ months, mixture = ~1 + months, random = ~1,
    subject = "id", classmb = ~pcl.bl, ng = 3, data = df, verbose = F,
    returndata = TRUE)

Statistical Model:
     Dataset: df
     Number of subjects: 4354
     Number of observations: 10814
     Number of observations deleted: 3886
     Number of latent classes: 3
```

```
     Number of parameters: 12

Iteration process:
     Convergence criteria satisfied
     Number of iterations:  1
     Convergence criteria: parameters= 2e-11
                         : likelihood= 5.8e-10
                         : second derivatives= 3.4e-11

Goodness-of-fit statistics:
     maximum log-likelihood: -44193.72
     AIC: 88411.44
     BIC: 88487.99

Maximum Likelihood Estimates:

Fixed effects in the class-membership model:
(the class of reference is the last class)

                     coef      Se    Wald p-value
intercept class1  0.34469 0.13350   2.582 0.00982
intercept class2  3.01771 0.09879  30.546 0.00000
pcl.bl class1    -0.00665 0.00352  -1.891 0.05862
pcl.bl class2    -0.06051 0.00362 -16.732 0.00000

Fixed effects in the longitudinal model:

                     coef      Se    Wald p-value
intercept class1 26.92831 1.08929  24.721 0.00000
intercept class2  9.91954 0.20771  47.757 0.00000
intercept class3 57.27827 0.97959  58.472 0.00000
months class1     2.26532 0.10887  20.808 0.00000
months class2    -0.24000 0.02528  -9.495 0.00000
months class3    -2.33187 0.13207 -17.657 0.00000

Variance-covariance matrix of the random-effects:
          intercept
intercept  39.97696

                             coef      Se
Residual standard error: 10.85302 0.10439

Be patient, grid search is running ...
Search completed, performing final estimation
Heterogenous linear mixed model
     fitted by maximum likelihood method

hlme(fixed = pcl ~ months, mixture = ~1 + months, random = ~1,
    subject = "id", classmb = ~pcl.bl, ng = 4, data = df, verbose = F,
    returndata = TRUE)
```

```
Statistical Model:
     Dataset: df
     Number of subjects: 4354
     Number of observations: 10814
     Number of observations deleted: 3886
     Number of latent classes: 4
     Number of parameters: 16

Iteration process:
     Convergence criteria satisfied
     Number of iterations:  1
     Convergence criteria: parameters= 7.7e-12
                         : likelihood= 1e-10
                         : second derivatives= 7.8e-12

Goodness-of-fit statistics:
     maximum log-likelihood: -43897.56
     AIC: 87827.13
     BIC: 87929.19

Maximum Likelihood Estimates:

Fixed effects in the class-membership model:
(the class of reference is the last class)

                      coef      Se    Wald p-value
intercept class1 -2.59871 0.09657 -26.911 0.00000
intercept class2 -3.80726 0.13953 -27.286 0.00000
intercept class3 -2.42347 0.08550 -28.344 0.00000
pcl.bl class1     0.05874 0.00429  13.679 0.00000
pcl.bl class2     0.08024 0.00453  17.715 0.00000
pcl.bl class3     0.05716 0.00425  13.452 0.00000

Fixed effects in the longitudinal model:

                      coef      Se    Wald p-value
intercept class1 42.51662 1.08083  39.337 0.00000
intercept class2 62.47446 1.21065  51.604 0.00000
intercept class3 21.11928 0.84371  25.031 0.00000
intercept class4  8.38637 0.21113  39.720 0.00000
months class1    -2.35248 0.11440 -20.564 0.00000
months class2    -0.09866 0.13978  -0.706 0.48028
months class3     2.02309 0.09769  20.708 0.00000
months class4    -0.20315 0.02639  -7.698 0.00000

Variance-covariance matrix of the random-effects:
          intercept
intercept  15.15611

                              coef      Se
Residual standard error: 10.62151 0.10316
```

```
Be patient, grid search is running ...
Search completed, performing final estimation
Heterogenous linear mixed model
     fitted by maximum likelihood method

hlme(fixed = pcl ~ months, mixture = ~1 + months, random = ~1,
    subject = "id", classmb = ~pcl.bl, ng = 5, data = df, verbose = F,
    returndata = TRUE)

Statistical Model:
     Dataset: df
     Number of subjects: 4354
     Number of observations: 10814
     Number of observations deleted: 3886
     Number of latent classes: 5
     Number of parameters: 20

Iteration process:
     Convergence criteria satisfied
     Number of iterations:  4
     Convergence criteria: parameters= 4.2e-13
                         : likelihood= 4.4e-11
                         : second derivatives= 2.6e-11

Goodness-of-fit statistics:
     maximum log-likelihood: -43772.34
     AIC: 87584.68
     BIC: 87712.25

Maximum Likelihood Estimates:

Fixed effects in the class-membership model:
(the class of reference is the last class)

                     coef      Se    Wald p-value
intercept class1 -2.49434 0.21686 -11.502 0.00000
intercept class2 -1.09668 0.13985  -7.842 0.00000
intercept class3  2.13552 0.09050  23.597 0.00000
intercept class4 -0.38875 0.13751  -2.827 0.00470
pcl.bl class1     0.03217 0.00505   6.373 0.00000
pcl.bl class2     0.01919 0.00422   4.552 0.00001
pcl.bl class3    -0.05633 0.00446 -12.628 0.00000
pcl.bl class4     0.00396 0.00449   0.883 0.37722

Fixed effects in the longitudinal model:

                     coef      Se    Wald p-value
intercept class1 71.07610 2.06095  34.487 0.00000
intercept class2 54.62429 1.07994  50.581 0.00000
intercept class3  7.18775 0.19994  35.950 0.00000
```

```
intercept class4 20.12952 1.12214  17.938 0.00000
intercept class5 30.56043 1.09067  28.020 0.00000
months class1     0.29383 0.18353   1.601 0.10938
months class2    -1.68616 0.25338  -6.655 0.00000
months class3    -0.13999 0.02705  -5.175 0.00000
months class4     2.32823 0.11450  20.333 0.00000
months class5    -1.46150 0.12659 -11.545 0.00000

Variance-covariance matrix of the random-effects:
          intercept
intercept         0

                             coef      Se
Residual standard error: 10.67058 0.08531

R version 4.1.0 (2021-05-18)
Platform: x86_64-pc-linux-gnu (64-bit)
Running under: CentOS Linux 7 (Core)

Matrix products: default
BLAS:   /sw/arcts/centos7/stacks/gcc/8.2.0/R/4.1.0/lib64/R/lib/libRbla
s.so
LAPACK:
/sw/arcts/centos7/stacks/gcc/8.2.0/R/4.1.0/lib64/R/lib/libRlapack.so

Random number generation:
 RNG:     L'Ecuyer-CMRG
 Normal:  Inversion
 Sample:  Rejection

locale:
 [1] LC_CTYPE=en_US.UTF-8       LC_NUMERIC=C
 [3] LC_TIME=en_US.UTF-8        LC_COLLATE=en_US.UTF-8
 [5] LC_MONETARY=en_US.UTF-8    LC_MESSAGES=en_US.UTF-8
 [7] LC_PAPER=en_US.UTF-8       LC_NAME=C
 [9] LC_ADDRESS=C               LC_TELEPHONE=C
[11] LC_MEASUREMENT=en_US.UTF-8 LC_IDENTIFICATION=C

attached base packages:
[1] parallel  stats     graphics  grDevices
utils     datasets  methods
[8] base

other attached packages:
[1] lcmm_1.9.3      mvtnorm_1.1-3   survival_3.2-11

loaded via a namespace (and not attached):
[1] compiler_4.1.0  Matrix_1.3-3    splines_4.1.0   grid_4.1.0
[5] lattice_0.20-44
```

Appendix 3. Analysis code and output of the LCGA models (fixed intercepts and slopes)

```
#Syntax
library(lcmm)
#1 trajectory
lcga1 <- hlme(pcl ~ months,  subject = "id", ng = 1, data = df,
verbose = F, returndata = TRUE)
summary(lcga1)
saveRDS(lcga1, "lcga1.prs.rds")

#2 trajectories
lcga2 <- gridsearch(rep = 100, maxiter = 100, minit = lcga1, cl = 16,
hlme(pcl ~ months,  subject = "id", ng = 2, data = df, mixture = ~1 +
months, verbose = F, returndata = TRUE, classmb = ~pcl.bl))
summary(lcga2)
saveRDS(lcga2, "lcga2.prs.rds")

#3 trajectories
lcga3 <- gridsearch(rep = 100, maxiter = 100, minit = lcga1, cl = 16,
hlme(pcl ~ months,  subject = "id", ng = 3, data = df, mixture = ~1 +
months, verbose = F, returndata = TRUE, classmb = ~pcl.bl))
summary(lcga3)
saveRDS(lcga3, "lcga3.prs.rds")

#4 trajectories
lcga4 <- gridsearch(rep = 100, maxiter = 100, minit = lcga1, cl = 16,
hlme(pcl ~ months,  subject = "id", ng = 4, data = df, mixture = ~1 +
months, verbose = F, returndata = TRUE, classmb = ~pcl.bl))
summary(lcga4)
saveRDS(lcga4, "lcga4.prs.rds")

#5 trajectories
lcga5 <- gridsearch(rep = 100, maxiter = 100, minit = lcga1, cl = 16,
hlme(pcl ~ months,  subject = "id", ng = 5, data = df, mixture = ~1 +
months, verbose = F, returndata = TRUE, classmb = ~pcl.bl))
summary(lcga5)
saveRDS(lcga5, "lcga5.prs.rds")
sessionInfo()

#Output
Heterogenous linear mixed model
     fitted by maximum likelihood method

hlme(fixed = pcl ~ months, subject = "id", ng = 1, data = df,
    verbose = F, returndata = TRUE)

Statistical Model:
     Dataset: df
     Number of subjects: 4355
     Number of observations: 10815
     Number of observations deleted: 3885
```

```
     Number of latent classes: 1
     Number of parameters: 3

Iteration process:
     Convergence criteria satisfied
     Number of iterations:  15
     Convergence criteria: parameters= 1.5e-09
                         : likelihood= 4.5e-08
                         : second derivatives= 9.6e-14

Goodness-of-fit statistics:
     maximum log-likelihood: -46794.13
     AIC: 93594.25
     BIC: 93613.39

Maximum Likelihood Estimates:

Fixed effects in the longitudinal model:

              coef      Se    Wald p-value
intercept 14.71941 0.23248  63.315 0.00000
months    -0.08219 0.03425  -2.400 0.01640

                             coef      Se
Residual standard error: 18.31722 0.12458

Be patient, grid search is running ...
Search completed, performing final estimation
Heterogenous linear mixed model
     fitted by maximum likelihood method

hlme(fixed = pcl ~ months, mixture = ~1 + months, subject = "id",
    classmb = ~pcl.bl, ng = 2, data = df, verbose = F, returndata =
    TRUE)

Statistical Model:
     Dataset: df
     Number of subjects: 4354
     Number of observations: 10814
     Number of observations deleted: 3886
     Number of latent classes: 2
     Number of parameters: 7

Iteration process:
     Convergence criteria satisfied
     Number of iterations:  1
     Convergence criteria: parameters= 2.3e-11
                         : likelihood= 2e-10
                         : second derivatives= 8.5e-11

Goodness-of-fit statistics:
```

```
     maximum log-likelihood: -44745.41
     AIC: 89504.83
     BIC: 89549.48

Maximum Likelihood Estimates:

Fixed effects in the class-membership model:
(the class of reference is the last class)

                     coef      Se    Wald p-value
intercept class1  2.28128 0.06422  35.521 0.00000
pcl.bl class1    -0.05555 0.00299 -18.557 0.00000

Fixed effects in the longitudinal model:

                     coef      Se    Wald p-value
intercept class1  9.90164 0.19867  49.840 0.00000
intercept class2 43.59696 0.58927  73.985 0.00000
months class1    -0.18620 0.02841  -6.554 0.00000
months class2     0.10830 0.07249   1.494 0.13520

                             coef      Se
Residual standard error: 13.34773 0.09549

Be patient, grid search is running ...
Search completed, performing final estimation
Heterogenous linear mixed model
     fitted by maximum likelihood method

hlme(fixed = pcl ~ months, mixture = ~1 + months, subject = "id",
    classmb = ~pcl.bl, ng = 3, data = df, verbose = F, returndata =
    TRUE)

Statistical Model:
     Dataset: df
     Number of subjects: 4354
     Number of observations: 10814
     Number of observations deleted: 3886
     Number of latent classes: 3
     Number of parameters: 11

Iteration process:
     Convergence criteria satisfied
     Number of iterations:  1
     Convergence criteria: parameters= 2.2e-11
                         : likelihood= 2.7e-10
                         : second derivatives= 3.1e-11

Goodness-of-fit statistics:
     maximum log-likelihood: -44321.82
     AIC: 88665.64
```

```
     BIC: 88735.8

Maximum Likelihood Estimates:

Fixed effects in the class-membership model:
(the class of reference is the last class)

                     coef      Se    Wald p-value
intercept class1 -1.95237 0.06682 -29.218 0.00000
intercept class2 -3.47052 0.11942 -29.063 0.00000
pcl.bl class1     0.05582 0.00365  15.280 0.00000
pcl.bl class2     0.07658 0.00410  18.673 0.00000

Fixed effects in the longitudinal model:

                     coef      Se    Wald p-value
intercept class1 29.61326 0.72118  41.062 0.00000
intercept class2 60.91559 1.08631  56.076 0.00000
intercept class3  8.54712 0.21041  40.620 0.00000
months class1     0.29339 0.09744   3.011 0.00260
months class2    -0.45301 0.16443  -2.755 0.00587
months class3    -0.22076 0.02831  -7.797 0.00000

                             coef      Se
Residual standard error: 12.13090 0.09173

Be patient, grid search is running ...
Search completed, performing final estimation
Heterogenous linear mixed model
     fitted by maximum likelihood method

hlme(fixed = pcl ~ months, mixture = ~1 + months, subject = "id",
    classmb = ~pcl.bl, ng = 4, data = df, verbose = F, returndata =
    TRUE)

Statistical Model:
     Dataset: df
     Number of subjects: 4354
     Number of observations: 10814
     Number of observations deleted: 3886
     Number of latent classes: 4
     Number of parameters: 15

Iteration process:
     Convergence criteria satisfied
     Number of iterations:  1
     Convergence criteria: parameters= 2.9e-12
                         : likelihood= 1.2e-10
                         : second derivatives= 1.6e-12

Goodness-of-fit statistics:
```

```
     maximum log-likelihood: -43929.83
     AIC: 87889.65
     BIC: 87985.34

Maximum Likelihood Estimates:

Fixed effects in the class-membership model:
(the class of reference is the last class)

                     coef      Se    Wald p-value
intercept class1 -3.60411 0.12491 -28.854 0.00000
intercept class2 -2.38148 0.08906 -26.739 0.00000
intercept class3 -2.43869 0.09612 -25.372 0.00000
pcl.bl class1     0.08074 0.00433  18.636 0.00000
pcl.bl class2     0.05680 0.00427  13.316 0.00000
pcl.bl class3     0.05699 0.00425  13.420 0.00000

Fixed effects in the longitudinal model:

                     coef      Se    Wald p-value
intercept class1 60.81923 0.99746  60.974 0.00000
intercept class2 20.78174 0.82317  25.246 0.00000
intercept class3 39.49918 1.06474  37.097 0.00000
intercept class4  7.91413 0.19767  40.036 0.00000
months class1    -0.18134 0.13544  -1.339 0.18059
months class2     1.94498 0.11083  17.549 0.00000
months class3    -2.12781 0.12321 -17.270 0.00000
months class4    -0.18365 0.02736  -6.713 0.00000

                             coef      Se
Residual standard error: 11.13166 0.08784

Be patient, grid search is running ...
Search completed, performing final estimation
Heterogenous linear mixed model
     fitted by maximum likelihood method

hlme(fixed = pcl ~ months, mixture = ~1 + months, subject = "id",
    classmb = ~pcl.bl, ng = 5, data = df, verbose = F, returndata =
    TRUE)

Statistical Model:
     Dataset: df
     Number of subjects: 4354
     Number of observations: 10814
     Number of observations deleted: 3886
     Number of latent classes: 5
     Number of parameters: 19

Iteration process:
     Convergence criteria satisfied
```

```
     Number of iterations:  1 
     Convergence criteria: parameters= 3.8e-11 
                         : likelihood= 3.6e-10 
                         : second derivatives= 2.7e-11 

Goodness-of-fit statistics: 
     maximum log-likelihood: -43772.34  
     AIC: 87582.68  
     BIC: 87703.87  


Maximum Likelihood Estimates: 

Fixed effects in the class-membership model:
(the class of reference is the last class) 

                     coef      Se    Wald p-value
intercept class1 -2.52427 0.09449 -26.715 0.00000
intercept class2 -2.13552 0.09033 -23.642 0.00000
intercept class3 -3.23220 0.12013 -26.907 0.00000
intercept class4 -4.62986 0.20519 -22.564 0.00000
pcl.bl class1     0.06030 0.00463  13.035 0.00000
pcl.bl class2     0.05633 0.00446  12.636 0.00000
pcl.bl class3     0.07552 0.00451  16.745 0.00000
pcl.bl class4     0.08851 0.00558  15.858 0.00000

Fixed effects in the longitudinal model:

                     coef      Se    Wald p-value
intercept class1 20.12951 1.12198  17.941 0.00000
intercept class2 30.56043 1.09001  28.037 0.00000
intercept class3 54.62429 1.08010  50.574 0.00000
intercept class4 71.07610 2.06283  34.456 0.00000
intercept class5  7.18775 0.19983  35.969 0.00000
months class1     2.32823 0.11443  20.346 0.00000
months class2    -1.46150 0.12645 -11.558 0.00000
months class3    -1.68616 0.25342  -6.654 0.00000
months class4     0.29383 0.18402   1.597 0.11032
months class5    -0.13999 0.02702  -5.181 0.00000

                             coef      Se
Residual standard error: 10.67058 0.08531

R version 4.1.0 (2021-05-18)
Platform: x86_64-pc-linux-gnu (64-bit)
Running under: CentOS Linux 7 (Core)

Matrix products: default
BLAS:   /sw/arcts/centos7/stacks/gcc/8.2.0/R/4.1.0/lib64/R/lib/libRbla
s.so
```

```
LAPACK:
/sw/arcts/centos7/stacks/gcc/8.2.0/R/4.1.0/lib64/R/lib/libRlapack.so

Random number generation:
 RNG:     L'Ecuyer-CMRG
 Normal:  Inversion
 Sample:  Rejection

locale:
 [1] LC_CTYPE=en_US.UTF-8       LC_NUMERIC=C
 [3] LC_TIME=en_US.UTF-8        LC_COLLATE=en_US.UTF-8
 [5] LC_MONETARY=en_US.UTF-8    LC_MESSAGES=en_US.UTF-8
 [7] LC_PAPER=en_US.UTF-8       LC_NAME=C
 [9] LC_ADDRESS=C               LC_TELEPHONE=C
[11] LC_MEASUREMENT=en_US.UTF-8 LC_IDENTIFICATION=C

attached base packages:
[1] parallel  stats     graphics  grDevices
utils     datasets  methods
[8] base

other attached packages:
[1] lcmm_1.9.3      mvtnorm_1.1-3   survival_3.2-11

loaded via a namespace (and not attached):
[1] compiler_4.1.0  Matrix_1.3-3    splines_4.1.0   grid_4.1.0
[5] lattice_0.20-44
```

Appendix 4. PRS associations with trajectories after controlling for additional variables suggested by reviewers

In our analyses of PRS associations with trajectories, we controlled for a total of 20 demographic, ancestral, and trauma-related variables (i.e., potentially traumatic experiences; PTEs). Reviewers suggested additional analyses controlling for length of deployment and disaggregating the types of PTEs from the pre-deployment PTE indicators that we used in our analyses. The resulting analyses controlled for 49 variables, including the original control variables, and the addition of length of deployment and 30 types of PTEs measured at pre-deployment (15 related to prior deployments such as combat, direct/indirect fire, getting wounded by the enemy, having a "close call", member of unit wounded or killed, direct responsibility for death of an enemy combatant, direct responsibility for non-combatant, direct responsibility for death of ally, saving the life of a soldier/civilian, seeing homes/villages destroyed, exposure to sights/sounds/smells of death and dying, witnessing violence toward locals/non-combatants, physical assault, sexual assault, bullied by members of unit; and 15 related to lifetime experiences such as physical assault, sexual assault, serious assault of friend/relative, murder of friend relative, suicide of friend/relative, attempted suicide of friend/relative, combat death of friend/relative, accidental death of friend/relative, witnessing someone being injured or killed, discovering/handling dead body, life threatening illness or injury, natural disaster where death was possible, other life-threatening experience, bullied during childhood/adolescence, and life-threatening experience of friend/relative.)

In the models that controlled for these additional covariates, there were some fluctuations in the *p*-values of PRS associations with trajectories: PTSD $p = .029$, MDD $p = .006$, AUD $p = .807$, neuroticism $p = .027$, schizophrenia $p = .919$, suicide attempt $p = .817$. There were also

some fluctuations in the adjusted odds ratios AOR for the PTSD and MDD PRS, though the pattern of results was similar and in some cases, the AOR were nearly identical.

	Original analyses controlling for 20 covariates	Additional analyses controlling for 49 covariates
PTSD-PRS		
High-Severity vs Increasing-Severity	1.09 [0.92, 1.30]	1.07 [0.89, 1.28]
High-Severity vs Decreasing-Severity	1.13 [0.94, 1.36]	1.11 [0.92, 1.33]
High-Severity vs Low-Severity	1.23 [1.06, 1.43]	1.19 [1.02, 1.40]
Increasing-Severity vs Decreasing-Severity	1.04 [0.89, 1.20]	1.04 [0.89, 1.21]
Increasing-Severity vs Low-Severity	1.12 [1.01, 1.25]	1.12 [1.01, 1.25]
Decreasing-Severity vs Low-Severity	1.08 [0.96, 1.22]	1.08 [0.96, 1.22]
MDD-PRS		
High-Severity vs Increasing-Severity	1.02 [0.86, 1.22]	1.02 [0.85, 1.22]
High-Severity vs Decreasing-Severity	1.02 [0.85, 1.22]	0.99 [0.83, 1.19]
High-Severity vs Low-Severity	1.18 [1.02, 1.37]	1.16 [0.99, 1.35]
Increasing-Severity vs Decreasing-Severity	0.99 [0.86, 1.15]	0.97 [0.84, 1.13]
Increasing-Severity vs Low-Severity	1.16 [1.04, 1.29]	1.14 [1.02, 1.27]
Decreasing-Severity vs Low-Severity	1.16 [1.03, 1.31]	1.17 [1.03, 1.32]
